# Supplementary material for: Pyruvate Dehydrogenase Kinase 1 inhibition mediated oxidative phosphorylation enhancement in cartilage promotes osteoarthritis progression
Source: BMC Musculoskelet Disord. 2023 Jul 20;24:597. doi: 10.1186/s12891-023-06585-6 (PMC10357736; doi:10.1186/s12891-023-06585-6)
Supplement: Supplementary file 1 — Additional file 1. [file 12891_2023_6585_MOESM1_ESM.zip › KEGG.Gsea.1653622432464/KEGG_CELL_ADHESION_MOLECULES_CAMS.html]

Details for gene set KEGG\_CELL\_ADHESION\_MOLECULES\_CAMS[GSEA]

|  || Dataset | 55457\_collapsed\_to\_symbols.cls.cls #OA\_versus\_normal.cls.cls #OA\_versus\_normal\_repos |
| Phenotype | cls.cls#OA\_versus\_normal\_repos |
| Upregulated in class | normal |
| GeneSet | KEGG\_CELL\_ADHESION\_MOLECULES\_CAMS |
| Enrichment Score (ES) | -0.28243813 |
| Normalized Enrichment Score (NES) | -1.2715582 |
| Nominal p-value | 0.06790123 |
| FDR q-value | 0.29922017 |
| FWER p-Value | 1.0 |
Table: GSEA Results Summary

  

Fig 1: Enrichment plot: KEGG\_CELL\_ADHESION\_MOLECULES\_CAMS      
 Profile of the Running ES Score & Positions of GeneSet Members on the Rank Ordered List

  

| SYMBOL | TITLE | RANK IN GENE LIST | RANK METRIC SCORE | RUNNING ES | CORE ENRICHMENT || 1 | CD58 | CD58 molecule [Source:HGNC Symbol;Acc:HGNC:1688] | 580 | 0.630 | -0.0260 | No |
| 2 | HLA-DMB | "major histocompatibility complex, class II, DM beta [Source:HGNC Symbol;Acc:HGNC:4935]" | 679 | 0.598 | -0.0152 | No |
| 3 | CD86 | CD86 molecule [Source:HGNC Symbol;Acc:HGNC:1705] | 689 | 0.596 | 0.0025 | No |
| 4 | ITGB2 | integrin subunit beta 2 [Source:HGNC Symbol;Acc:HGNC:6155] | 827 | 0.562 | 0.0091 | No |
| 5 | CD2 | CD2 molecule [Source:HGNC Symbol;Acc:HGNC:1639] | 1306 | 0.457 | -0.0142 | No |
| 6 | HLA-DQA1 | "major histocompatibility complex, class II, DQ alpha 1 [Source:HGNC Symbol;Acc:HGNC:4942]" | 1334 | 0.452 | -0.0024 | No |
| 7 | PDCD1LG2 | programmed cell death 1 ligand 2 [Source:HGNC Symbol;Acc:HGNC:18731] | 1378 | 0.446 | 0.0080 | No |
| 8 | ALCAM | activated leukocyte cell adhesion molecule [Source:HGNC Symbol;Acc:HGNC:400] | 1513 | 0.427 | 0.0107 | No |
| 9 | MPZL1 | myelin protein zero like 1 [Source:HGNC Symbol;Acc:HGNC:7226] | 1712 | 0.396 | 0.0074 | No |
| 10 | CLDN4 | claudin 4 [Source:HGNC Symbol;Acc:HGNC:2046] | 1716 | 0.395 | 0.0193 | No |
| 11 | HLA-DMA | "major histocompatibility complex, class II, DM alpha [Source:HGNC Symbol;Acc:HGNC:4934]" | 1769 | 0.388 | 0.0272 | No |
| 12 | ITGA4 | integrin subunit alpha 4 [Source:HGNC Symbol;Acc:HGNC:6140] | 1854 | 0.379 | 0.0323 | No |
| 13 | ICAM2 | intercellular adhesion molecule 2 [Source:HGNC Symbol;Acc:HGNC:5345] | 2048 | 0.356 | 0.0282 | No |
| 14 | NCAM2 | neural cell adhesion molecule 2 [Source:HGNC Symbol;Acc:HGNC:7657] | 2085 | 0.352 | 0.0362 | No |
| 15 | HLA-DOB | "major histocompatibility complex, class II, DO beta [Source:HGNC Symbol;Acc:HGNC:4937]" | 2147 | 0.344 | 0.0421 | No |
| 16 | CDH1 | cadherin 1 [Source:HGNC Symbol;Acc:HGNC:1748] | 2589 | 0.292 | 0.0166 | No |
| 17 | CDH2 | cadherin 2 [Source:HGNC Symbol;Acc:HGNC:1759] | 2679 | 0.282 | 0.0183 | No |
| 18 | HLA-DQB1 | "major histocompatibility complex, class II, DQ beta 1 [Source:HGNC Symbol;Acc:HGNC:4944]" | 2840 | 0.266 | 0.0140 | No |
| 19 | SDC2 | syndecan 2 [Source:HGNC Symbol;Acc:HGNC:10659] | 2880 | 0.262 | 0.0190 | No |
| 20 | CLDN1 | claudin 1 [Source:HGNC Symbol;Acc:HGNC:2032] | 3089 | 0.241 | 0.0102 | No |
| 21 | SIGLEC1 | sialic acid binding Ig like lectin 1 [Source:HGNC Symbol;Acc:HGNC:11127] | 3124 | 0.237 | 0.0148 | No |
| 22 | NRCAM | neuronal cell adhesion molecule [Source:HGNC Symbol;Acc:HGNC:7994] | 3260 | 0.225 | 0.0112 | No |
| 23 | NLGN4X | neuroligin 4 X-linked [Source:HGNC Symbol;Acc:HGNC:14287] | 3974 | 0.158 | -0.0397 | No |
| 24 | CD8B | CD8b molecule [Source:HGNC Symbol;Acc:HGNC:1707] | 4226 | 0.135 | -0.0552 | No |
| 25 | CADM1 | cell adhesion molecule 1 [Source:HGNC Symbol;Acc:HGNC:5951] | 4259 | 0.132 | -0.0536 | No |
| 26 | CD22 | CD22 molecule [Source:HGNC Symbol;Acc:HGNC:1643] | 4296 | 0.128 | -0.0525 | No |
| 27 | CD34 | CD34 molecule [Source:HGNC Symbol;Acc:HGNC:1662] | 4341 | 0.125 | -0.0521 | No |
| 28 | HLA-DOA | "major histocompatibility complex, class II, DO alpha [Source:HGNC Symbol;Acc:HGNC:4936]" | 4344 | 0.125 | -0.0484 | No |
| 29 | CD4 | CD4 molecule [Source:HGNC Symbol;Acc:HGNC:1678] | 4465 | 0.116 | -0.0542 | No |
| 30 | CD28 | CD28 molecule [Source:HGNC Symbol;Acc:HGNC:1653] | 4543 | 0.110 | -0.0569 | No |
| 31 | SELP | selectin P [Source:HGNC Symbol;Acc:HGNC:10721] | 4573 | 0.107 | -0.0558 | No |
| 32 | SELPLG | selectin P ligand [Source:HGNC Symbol;Acc:HGNC:10722] | 4816 | 0.086 | -0.0721 | No |
| 33 | NEO1 | neogenin 1 [Source:HGNC Symbol;Acc:HGNC:7754] | 4826 | 0.085 | -0.0702 | No |
| 34 | PTPRF | protein tyrosine phosphatase receptor type F [Source:HGNC Symbol;Acc:HGNC:9670] | 4839 | 0.084 | -0.0685 | No |
| 35 | CADM3 | cell adhesion molecule 3 [Source:HGNC Symbol;Acc:HGNC:17601] | 5032 | 0.070 | -0.0814 | No |
| 36 | SDC3 | syndecan 3 [Source:HGNC Symbol;Acc:HGNC:10660] | 5114 | 0.063 | -0.0858 | No |
| 37 | CDH4 | cadherin 4 [Source:HGNC Symbol;Acc:HGNC:1763] | 5121 | 0.062 | -0.0843 | No |
| 38 | CNTNAP2 | contactin associated protein 2 [Source:HGNC Symbol;Acc:HGNC:13830] | 5188 | 0.057 | -0.0878 | No |
| 39 | NRXN3 | neurexin 3 [Source:HGNC Symbol;Acc:HGNC:8010] | 5269 | 0.050 | -0.0925 | No |
| 40 | VCAM1 | vascular cell adhesion molecule 1 [Source:HGNC Symbol;Acc:HGNC:12663] | 5283 | 0.049 | -0.0920 | No |
| 41 | CLDN10 | claudin 10 [Source:HGNC Symbol;Acc:HGNC:2033] | 5479 | 0.034 | -0.1062 | No |
| 42 | HLA-DPB1 | "major histocompatibility complex, class II, DP beta 1 [Source:HGNC Symbol;Acc:HGNC:4940]" | 5486 | 0.033 | -0.1056 | No |
| 43 | L1CAM | L1 cell adhesion molecule [Source:HGNC Symbol;Acc:HGNC:6470] | 5512 | 0.032 | -0.1066 | No |
| 44 | SDC1 | syndecan 1 [Source:HGNC Symbol;Acc:HGNC:10658] | 5526 | 0.031 | -0.1067 | No |
| 45 | CD40LG | CD40 ligand [Source:HGNC Symbol;Acc:HGNC:11935] | 5529 | 0.030 | -0.1059 | No |
| 46 | JAM2 | junctional adhesion molecule 2 [Source:HGNC Symbol;Acc:HGNC:14686] | 5602 | 0.024 | -0.1108 | No |
| 47 | ITGAM | integrin subunit alpha M [Source:HGNC Symbol;Acc:HGNC:6149] | 5820 | 0.007 | -0.1276 | No |
| 48 | CLDN17 | claudin 17 [Source:HGNC Symbol;Acc:HGNC:2038] | 5931 | 0.000 | -0.1362 | No |
| 49 | CD6 | CD6 molecule [Source:HGNC Symbol;Acc:HGNC:1691] | 6121 | -0.014 | -0.1505 | No |
| 50 | ICOSLG | inducible T cell costimulator ligand [Source:HGNC Symbol;Acc:HGNC:17087] | 6128 | -0.015 | -0.1505 | No |
| 51 | NLGN3 | neuroligin 3 [Source:HGNC Symbol;Acc:HGNC:14289] | 6371 | -0.033 | -0.1684 | No |
| 52 | NRXN2 | neurexin 2 [Source:HGNC Symbol;Acc:HGNC:8009] | 6484 | -0.041 | -0.1759 | No |
| 53 | VCAN | versican [Source:HGNC Symbol;Acc:HGNC:2464] | 6546 | -0.045 | -0.1793 | No |
| 54 | HLA-DRA | "major histocompatibility complex, class II, DR alpha [Source:HGNC Symbol;Acc:HGNC:4947]" | 6550 | -0.046 | -0.1781 | No |
| 55 | ICAM3 | intercellular adhesion molecule 3 [Source:HGNC Symbol;Acc:HGNC:5346] | 6573 | -0.047 | -0.1784 | No |
| 56 | CD8A | CD8a molecule [Source:HGNC Symbol;Acc:HGNC:1706] | 6579 | -0.048 | -0.1773 | No |
| 57 | NECTIN3 | nectin cell adhesion molecule 3 [Source:HGNC Symbol;Acc:HGNC:17664] | 6767 | -0.061 | -0.1901 | No |
| 58 | CD40 | CD40 molecule [Source:HGNC Symbol;Acc:HGNC:11919] | 6825 | -0.065 | -0.1925 | No |
| 59 | CD80 | CD80 molecule [Source:HGNC Symbol;Acc:HGNC:1700] | 6918 | -0.074 | -0.1975 | No |
| 60 | NCAM1 | neural cell adhesion molecule 1 [Source:HGNC Symbol;Acc:HGNC:7656] | 7012 | -0.081 | -0.2023 | No |
| 61 | ITGAV | integrin subunit alpha V [Source:HGNC Symbol;Acc:HGNC:6150] | 7067 | -0.085 | -0.2039 | No |
| 62 | HLA-DRB1 | "major histocompatibility complex, class II, DR beta 1 [Source:HGNC Symbol;Acc:HGNC:4948]" | 7087 | -0.086 | -0.2027 | No |
| 63 | HLA-F | "major histocompatibility complex, class I, F [Source:HGNC Symbol;Acc:HGNC:4963]" | 7196 | -0.096 | -0.2082 | No |
| 64 | CLDN8 | claudin 8 [Source:HGNC Symbol;Acc:HGNC:2050] | 7517 | -0.120 | -0.2295 | No |
| 65 | NLGN1 | neuroligin 1 [Source:HGNC Symbol;Acc:HGNC:14291] | 7602 | -0.127 | -0.2322 | No |
| 66 | PTPRC | protein tyrosine phosphatase receptor type C [Source:HGNC Symbol;Acc:HGNC:9666] | 7744 | -0.137 | -0.2389 | No |
| 67 | ITGA9 | integrin subunit alpha 9 [Source:HGNC Symbol;Acc:HGNC:6145] | 7785 | -0.140 | -0.2378 | No |
| 68 | ITGB7 | integrin subunit beta 7 [Source:HGNC Symbol;Acc:HGNC:6162] | 7873 | -0.146 | -0.2401 | No |
| 69 | CLDN6 | claudin 6 [Source:HGNC Symbol;Acc:HGNC:2048] | 7883 | -0.147 | -0.2362 | No |
| 70 | CDH3 | cadherin 3 [Source:HGNC Symbol;Acc:HGNC:1762] | 7924 | -0.149 | -0.2348 | No |
| 71 | CD226 | CD226 molecule [Source:HGNC Symbol;Acc:HGNC:16961] | 7930 | -0.149 | -0.2306 | No |
| 72 | CNTN1 | contactin 1 [Source:HGNC Symbol;Acc:HGNC:2171] | 8095 | -0.163 | -0.2384 | No |
| 73 | SELL | selectin L [Source:HGNC Symbol;Acc:HGNC:10720] | 8319 | -0.179 | -0.2503 | No |
| 74 | NFASC | neurofascin [Source:HGNC Symbol;Acc:HGNC:29866] | 8525 | -0.196 | -0.2603 | No |
| 75 | CNTNAP1 | contactin associated protein 1 [Source:HGNC Symbol;Acc:HGNC:8011] | 8809 | -0.218 | -0.2757 | Yes |
| 76 | CLDN11 | claudin 11 [Source:HGNC Symbol;Acc:HGNC:8514] | 8817 | -0.219 | -0.2695 | Yes |
| 77 | MADCAM1 | mucosal vascular addressin cell adhesion molecule 1 [Source:HGNC Symbol;Acc:HGNC:6765] | 8908 | -0.225 | -0.2696 | Yes |
| 78 | CLDN14 | claudin 14 [Source:HGNC Symbol;Acc:HGNC:2035] | 9033 | -0.234 | -0.2721 | Yes |
| 79 | ITGA6 | integrin subunit alpha 6 [Source:HGNC Symbol;Acc:HGNC:6142] | 9112 | -0.240 | -0.2708 | Yes |
| 80 | CLDN9 | claudin 9 [Source:HGNC Symbol;Acc:HGNC:2051] | 9203 | -0.248 | -0.2702 | Yes |
| 81 | GLG1 | golgi glycoprotein 1 [Source:HGNC Symbol;Acc:HGNC:4316] | 9313 | -0.256 | -0.2708 | Yes |
| 82 | JAM3 | junctional adhesion molecule 3 [Source:HGNC Symbol;Acc:HGNC:15532] | 9376 | -0.262 | -0.2676 | Yes |
| 83 | CDH15 | cadherin 15 [Source:HGNC Symbol;Acc:HGNC:1754] | 9379 | -0.262 | -0.2597 | Yes |
| 84 | ITGB8 | integrin subunit beta 8 [Source:HGNC Symbol;Acc:HGNC:6163] | 9442 | -0.268 | -0.2563 | Yes |
| 85 | HLA-DPA1 | "major histocompatibility complex, class II, DP alpha 1 [Source:HGNC Symbol;Acc:HGNC:4938]" | 9481 | -0.273 | -0.2508 | Yes |
| 86 | MAG | myelin associated glycoprotein [Source:HGNC Symbol;Acc:HGNC:6783] | 9490 | -0.273 | -0.2430 | Yes |
| 87 | ITGAL | integrin subunit alpha L [Source:HGNC Symbol;Acc:HGNC:6148] | 9526 | -0.276 | -0.2373 | Yes |
| 88 | ICAM1 | intercellular adhesion molecule 1 [Source:HGNC Symbol;Acc:HGNC:5344] | 9740 | -0.296 | -0.2448 | Yes |
| 89 | F11R | F11 receptor [Source:HGNC Symbol;Acc:HGNC:14685] | 9765 | -0.297 | -0.2375 | Yes |
| 90 | CLDN5 | claudin 5 [Source:HGNC Symbol;Acc:HGNC:2047] | 9816 | -0.302 | -0.2321 | Yes |
| 91 | CDH5 | cadherin 5 [Source:HGNC Symbol;Acc:HGNC:1764] | 9946 | -0.316 | -0.2325 | Yes |
| 92 | NECTIN2 | nectin cell adhesion molecule 2 [Source:HGNC Symbol;Acc:HGNC:9707] | 10204 | -0.341 | -0.2421 | Yes |
| 93 | CD99 | CD99 molecule (Xg blood group) [Source:HGNC Symbol;Acc:HGNC:7082] | 10229 | -0.344 | -0.2333 | Yes |
| 94 | PECAM1 | platelet and endothelial cell adhesion molecule 1 [Source:HGNC Symbol;Acc:HGNC:8823] | 10304 | -0.351 | -0.2283 | Yes |
| 95 | ICOS | inducible T cell costimulator [Source:HGNC Symbol;Acc:HGNC:5351] | 10320 | -0.353 | -0.2186 | Yes |
| 96 | NRXN1 | neurexin 1 [Source:HGNC Symbol;Acc:HGNC:8008] | 10581 | -0.382 | -0.2272 | Yes |
| 97 | PDCD1 | programmed cell death 1 [Source:HGNC Symbol;Acc:HGNC:8760] | 10584 | -0.382 | -0.2156 | Yes |
| 98 | CTLA4 | cytotoxic T-lymphocyte associated protein 4 [Source:HGNC Symbol;Acc:HGNC:2505] | 10739 | -0.398 | -0.2153 | Yes |
| 99 | NECTIN1 | nectin cell adhesion molecule 1 [Source:HGNC Symbol;Acc:HGNC:9706] | 10771 | -0.402 | -0.2054 | Yes |
| 100 | HLA-G | "major histocompatibility complex, class I, G [Source:HGNC Symbol;Acc:HGNC:4964]" | 10869 | -0.413 | -0.2003 | Yes |
| 101 | ITGA8 | integrin subunit alpha 8 [Source:HGNC Symbol;Acc:HGNC:6144] | 11046 | -0.437 | -0.2006 | Yes |
| 102 | CLDN16 | claudin 16 [Source:HGNC Symbol;Acc:HGNC:2037] | 11104 | -0.444 | -0.1913 | Yes |
| 103 | MPZ | myelin protein zero [Source:HGNC Symbol;Acc:HGNC:7225] | 11151 | -0.451 | -0.1810 | Yes |
| 104 | CLDN7 | claudin 7 [Source:HGNC Symbol;Acc:HGNC:2049] | 11178 | -0.454 | -0.1691 | Yes |
| 105 | PTPRM | protein tyrosine phosphatase receptor type M [Source:HGNC Symbol;Acc:HGNC:9675] | 11289 | -0.470 | -0.1632 | Yes |
| 106 | HLA-E | "major histocompatibility complex, class I, E [Source:HGNC Symbol;Acc:HGNC:4962]" | 11659 | -0.526 | -0.1758 | Yes |
| 107 | CLDN15 | claudin 15 [Source:HGNC Symbol;Acc:HGNC:2036] | 11849 | -0.559 | -0.1734 | Yes |
| 108 | CNTN2 | contactin 2 [Source:HGNC Symbol;Acc:HGNC:2172] | 11858 | -0.561 | -0.1567 | Yes |
| 109 | SDC4 | syndecan 4 [Source:HGNC Symbol;Acc:HGNC:10661] | 12038 | -0.603 | -0.1521 | Yes |
| 110 | CLDN18 | claudin 18 [Source:HGNC Symbol;Acc:HGNC:2039] | 12254 | -0.662 | -0.1486 | Yes |
| 111 | SPN | sialophorin [Source:HGNC Symbol;Acc:HGNC:11249] | 12487 | -0.742 | -0.1438 | Yes |
| 112 | SELE | selectin E [Source:HGNC Symbol;Acc:HGNC:10718] | 12498 | -0.748 | -0.1216 | Yes |
| 113 | HLA-A | "major histocompatibility complex, class I, A [Source:HGNC Symbol;Acc:HGNC:4931]" | 12502 | -0.749 | -0.0987 | Yes |
| 114 | HLA-B | "major histocompatibility complex, class I, B [Source:HGNC Symbol;Acc:HGNC:4932]" | 12534 | -0.764 | -0.0776 | Yes |
| 115 | ITGB1 | integrin subunit beta 1 [Source:HGNC Symbol;Acc:HGNC:6153] | 12566 | -0.780 | -0.0560 | Yes |
| 116 | CLDN3 | claudin 3 [Source:HGNC Symbol;Acc:HGNC:2045] | 12615 | -0.807 | -0.0349 | Yes |
| 117 | HLA-DRB4 | "major histocompatibility complex, class II, DR beta 4 [Source:HGNC Symbol;Acc:HGNC:4952]" | 12636 | -0.815 | -0.0113 | Yes |
| 118 | PVR | PVR cell adhesion molecule [Source:HGNC Symbol;Acc:HGNC:9705] | 12831 | -1.035 | 0.0054 | Yes |
Table: GSEA details [plain text format]

  

Fig 2: KEGG\_CELL\_ADHESION\_MOLECULES\_CAMS      
 Blue-Pink O' Gram in the Space of the Analyzed GeneSet

  

Fig 3: KEGG\_CELL\_ADHESION\_MOLECULES\_CAMS: Random ES distribution      
 Gene set null distribution of ES for **KEGG\_CELL\_ADHESION\_MOLECULES\_CAMS**
